# A Sparse Bayesian Approach for Forward-Looking Superresolution Radar Imaging

**DOI:** 10.3390/s17061353

**Published:** 2017-06-10

**Authors:** Yin Zhang, Yongchao Zhang, Yulin Huang, Jianyu Yang

**Affiliations:** University of Electronic Science and Technology of China, Chengdu 610051, China; zhang_yongchao1@163.com (Y.Z.); yulinhuang@uestc.edu.cn (Y.H.); jyyang@uestc.edu.cn (J.Y.)

**Keywords:** forward-looking imaging, Bayesian criterion, sparse regularization

## Abstract

This paper presents a sparse superresolution approach for high cross-range resolution imaging of forward-looking scanning radar based on the Bayesian criterion. First, a novel forward-looking signal model is established as the product of the measurement matrix and the cross-range target distribution, which is more accurate than the conventional convolution model. Then, based on the Bayesian criterion, the widely-used sparse regularization is considered as the penalty term to recover the target distribution. The derivation of the cost function is described, and finally, an iterative expression for minimizing this function is presented. Alternatively, this paper discusses how to estimate the single parameter of Gaussian noise. With the advantage of a more accurate model, the proposed sparse Bayesian approach enjoys a lower model error. Meanwhile, when compared with the conventional superresolution methods, the proposed approach shows high cross-range resolution and small location error. The superresolution results for the simulated point target, scene data, and real measured data are presented to demonstrate the superior performance of the proposed approach.

## 1. Introduction

Forward-looking radar imaging with high cross-range resolution is of great significance for many civil and military applications [1,2]. Regrettably, the principle of a classic monostatic synthetic aperture radar (SAR) or Doppler beam sharpening (DBS) prevents high-resolution imaging in the forward-looking direction. The reason for this resolution problem is that terrain points situated symmetrically about the flight path have the same Doppler history, and the gradient of the Doppler frequency is small in the flight direction.

Many approaches are proposed for solving this low resolution problem. Monopulse and clean techniques are the beam sharpening techniques that could realize high target measurement accuracy [3,4,5]. However, this kind of method has serious location error when more than one target is distributed in one beam. Bistatic SAR is proposed to overcome the forward-looking imaging problem with a specific geometric structure. However, the complicated structure and synchronization problem are hardly solved [6,7,8,9].

Beccause the cross-range echo can be expressed by a linear equation system in the time domain, one may reconstruct the ground truth by solving the equation system. Inverse filter approaches have been presented, and achieve the improvements of cross-range resolution on a high signal-to-noise ratio (SNR) [10,11]. However, the linear equation system is typically ill-conditioned because of the low-pass property of the antenna pattern, and the solution of the associated system can cause noise amplification and blurring effects. In [12,13,14], a scheme of the truncated singular value decomposition (TSVD) method was proposed by rejecting the small singular values. The method was demonstrated to significantly suppress the noise. However, this advantage is at the cost of limited resolution improvement. In addition, the number of reserved truncated values—which plays the role of the regularization parameter—is difficult to determine.

Recently, the Bayesian deconvolution approach has been developed for high-resolution radar imaging [15,16,17,18]. It can also be employed to deal with the forward-looking imaging problem model because the received signal of the forward-looking scanning radar in the cross-range dimension can be regarded as the convolution of the antenna pattern and the ground truth [19,20,21]. As will be demonstrated in this paper, the Bayesian deconvolution approach can provide much higher resolution than the real beam image. However, the target location error appears, and the noise is amplified severely. This is because, firstly, in most of the traditional Bayesian deconvolution approaches, they compute the maximum a posteriori estimation based only on the amplitude statistics property of received echo for calculation convenience. Therefore, the added phase caused by the platform movement will certainly lead to the error of recovery result. Secondly, without reasonable prior information about the practical applications of forward-looking imaging, the false targets that result from the noise amplification seriously influence the imaging performance.

In this paper, the received echo of forward-looking scanning radar in the cross-range dimension is deduced first. We derive a novel signal model for forward-looking radar imaging. Different from the conventional convolution model in the cross-range dimension, the proposed model will consider the effect of Doppler history, and will be more accurate. Then, the Gaussian-based modeling of the noise is considered in the proposed method, which has been investigated and successfully applied to synthetic aperture radar imaging and multiple-input multiple-output (MIMO) radar imaging [22,23,24,25]. Compared to the traditional statistical hypothesis, the Gaussian distribution is often more accurate for modeling the characteristics of noise in radar imaging. Moreover, high accuracy superresolution results can be obtained by using the complex signal recovery deconvolution because no modulus or approximate operators are needed.

Then, based on the model, the $l_{p}$-norm sparse constraint is considered as the prior information to reconstruct the target distribution. The forward-looking imaging is often applied to the important ground and sea surface target location and tracing, obstacle avoidance of helicopters, etc. The targets of interest we often focus on are sparse for the whole imaging region. The problem of reconstruction for sparse targets has been deeply discussed, and several sparse approaches have been developed in array signal processing, SAR and Inverse SAR (ISAR) imaging, etc. [26,27,28,29]. Besides, the existing sparse methods demonstrate that the sparsity constraints also have denoising capability [30,31]. With these motivations, we develop a sparse Bayesian method by adopting the $l_{p}$-norm (0<p≤1) sparse constraint. The advantage of the $l_{p}$-norm has been discussed in the related work on the sparse learning via iterative minimization (SLIM) method [23]. It shows that the $l_{p}$-norm could realize a high sparse result compared to the traditional $l_{1}$-norm [23,32], which intends to provide higher resolution and reject false targets. With an accurate signal model and prior information, the proposed sparse Bayesian approach provides better superresolution performance than the conventional Bayesian approaches.

This paper is organized as follows. In Section 2, the received signal model for the forward-looking scanning radar is constructed. Then, some techniques are taken to preprocess the received signal. We describe the forward-looking signal model of forward-looking scanning radar as a matrix operation. In Section 3, the likelihood function and prior knowledge are combined to make the objective function converge to a suitable solution. In addition, the derivation of the objective function is provided. In Section 4, the simulations and real measured data are given to show the superresolution performance of the proposed algorithms. Conclusions are provided in Section 5.

## 2. Signal Model of the Forward-Looking Scanning Radar Image

**Figure 1:**
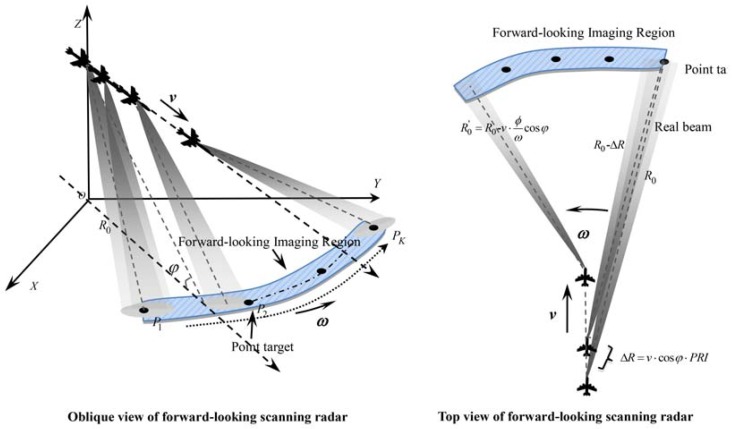
Geometric model of forward-looking scanning radar.

Figure 1 shows the geometric model of the forward-looking scanning radar. The antenna sweeps across the forward-looking scene with counterclockwise mechanical movement. The airborne platform moves along the radial direction with a fixed velocity υ, and the platform height is *H*. Suppose some targets are distributed in the forward-looking imaging region; target $P_{i}$ is taken as an example to analyze the range history between the point target and sensor. The original distance between the sensor and target $P_{i}$ is $R_{0}$. The angle of the radial direction of $P_{i}$ is $\theta_{i}$, and φ is the incident angle of the antenna. The range history between $P_{i}$ and the antenna is:

(1) $$R\left(t\right)=\sqrt{R_{0}^{2}+{\left(\upsilon t\right)}^{2}-2R_{0}\cos\theta_{i}\cos\varphi \upsilon t}.$$

which can be expanded into a Taylor series as:

(2) $$R\left(t\right)=R_{0}-\upsilon \cos\theta_{i}\cos\varphi t+\frac{\upsilon^{2}\left(1-\cos\theta_{i}^{2}\cos\varphi^{2}\right)}{2R_{0}}t^{2}+O\left(t^{2}\right).$$

In side-looking mode, θ is within a small range around $90^{\circ }$, and the quadratic terms in (2) play the dominant role. Then, the resolution can be improved significantly via the SAR technique [33,34]. However, in forward-looking mode, θ is within a small range around $0^{\circ }$, and the linear term in (2) plays the dominant role. In this case, the Doppler bandwidth becomes much smaller, and SAR cannot work for forward-looking imaging.

Since the second-order term above is very small and the product of time and velocity is also much smaller than $R_{0}$, Equation (2) can be approximated as:

(3) $$R\left(t\right)≅R_{0}-\cos\theta_{i}\cos\varphi \upsilon t.$$

Equation (3) shows that targets in different cross-range angles of the forward-looking region have different range histories, which result in a different added Doppler phase of different targets. This is why we cannot employ real-valued signal recovery approaches to deal with the forward-looking imaging problem. In forward-looking imaging, we also transmit linear frequency modulation (LFM) signals in order to easily realize high range resolution. For far distance imaging regions, the signal received after the antenna scans in the forward-looking region is:

(4) $$g\left(t,\tau \right)=\sum_{k=1}^{K}\sigma_{k}a\left(\theta_{k},\tau \right)rect\left(\frac{\tau -\frac{2R\left(t\right)}{c}}{T}\right)\exp\left(-j\frac{4\pi }{\lambda }R\left(t\right)\right)\exp\left(j\pi K_{r}{\left[\tau -\frac{2R\left(t\right)}{c}\right]}^{2}\right)$$

where *K* is the number of samples in the cross-range dimension that can be calculated by K=ϕ·PRF/ω, wherein ϕ is the scanning region, ω is the sweep speed, and PRF is the pulse repetition frequency. $rect\left(\cdot \right)$ denotes the unit rectangular function, and *T* denotes the signal duration. $rect\left(\frac{t}{T}\right)=1$ when $\left|t\right|\leq T$. *t* and τ represent the fast and slow time in the range dimension and cross-range dimension, respectively. In radar signal processing, operations that are applied to data from a single pulse occur on the shortest time scale (often referred to as fast time), and the sampling interval between pulses is referred to as slow time. $f_{c}$ denotes carrier frequency, and $K_{r}$ denotes the chirp rate. a(·) is the antenna pattern modulation function, and $\theta_{k}$ denotes the antenna pointing direction of the k-th signal.

Because the transmitting signals are LFM signals, high range resolution can be realized by matched filtering. After this operation, the received signal becomes:

(5) $$\begin{array}{c} g\left(t,\tau \right)=\sum_{k=1}^{K}\sigma_{k}a\left(\theta_{k},\tau \right)\sin c\left[B\left(\tau -\frac{2R\left(t\right)}{c}\right)\right]\exp\left(-j\frac{4\pi }{\lambda }R\left(t\right)\right). \end{array}$$

where *B* is the bandwidth of transmitted signals. The range migration adjusting function is constructed to eliminate the effect of movement. The two-dimensional received signal is then:

(6) $$\begin{array}{c} g\left(t,\tau \right)=\sum_{k=1}^{K}\sigma_{k}a\left(\theta_{k},\tau \right)\sin c\left[B\left(\tau -\frac{2R_{0}}{c}\right)\right]\exp\left(-j\frac{4\pi }{\lambda }R\left(t\right)\right). \end{array}$$

The preprocessed signal has high range resolution and low cross-range resolution. Meanwhile, the traditional matched filtering technique cannot be applied to the cross-range dimension of the forward-looking region because of the limited Doppler bandwidth. On the other hand, the cross-range received signal can be regarded as the convolution of the target scattering coefficient and antenna pattern; therefore, we can employ deconvolution approaches to improve cross-range resolution. For mathematical simplicity, the discrete received signal is rearranged and written as the matrix and vector form:

(7) $$\begin{array}{c} g=\mathrm{Ax}+n=\left[\begin{array}{ccc} A_{M\times K} & & \\ & \ddots & \\ & & A_{M\times K} \end{array}\right]\left[\begin{array}{c} \begin{array}{c} x\left(1,\;1\right)\\ x\left(1,\;2\right) \end{array}\\ \vdots \\ \begin{array}{c} x\left(1,\;K\right)\\ \vdots \\ x\left(N,\;K\right) \end{array} \end{array}\right]+n \end{array}$$

where $g={\left[g\left(1,\;1\right),g\left(1,\;2\right),\cdots ,g\left(1,\;M\right),\cdots ,g\left(N,\;M\right)\right]}^{T}$ represents the measurement range profiles, which were rearranged in the cross-range dimension with size NM×1. *N* and *M* are the discrete sampling numbers of the received signal in range dimension and cross-range dimension, respectively. $x={\left[x\left(1,\;1\right),x\left(1,\;2\right),\cdots ,x\left(1,\;K\right),\cdots ,x\left(N,\;K\right)\right]}^{T}$ represents the unknown scene amplitude profiles, which were rearranged in the cross-range dimension with size NK×1. n is the noise vector with dimension NM×1, which satisfies the complex Gaussian distribution. The matrix A is the measurement matrix with size NM×NK, which was composed of the convolution matrix $A_{M\times K}$. $A_{M\times K}=\left[a_{1},\;a_{2},\;\cdots ,\;a_{K}\right]$ is the measurement matrix of the single cross-range profile, which is given by the following equation.

$$\left(\begin{array}{cccc} a_{1} & 0 & \cdots & 0\\ a_{2}\cdot e^{-j\frac{4\pi }{\lambda }\cdot \cos\theta_{1}\cos\varphi \cdot \upsilon \cdot PRI} & a_{1}\cdot e^{-j\frac{4\pi }{\lambda }\cdot \cos\theta_{2}\cos\varphi \cdot \upsilon \cdot PRI} & & \vdots \\ \vdots & \vdots & \vdots & \vdots \\ \begin{array}{c} a_{N_{b}}\cdot e^{-j\frac{4\pi }{\lambda }\cdot \cos\theta_{1}\cos\varphi \cdot \upsilon \cdot \left(N_{b}-1\right)\cdot PRI}\\ 0\\ \vdots \\ \vdots \\ \vdots \\ 0 \end{array} & \begin{array}{c} a_{N_{b}-1}\cdot e^{-j\frac{4\pi }{\lambda }\cdot \cos\theta_{2}\cos\varphi \cdot \upsilon \cdot \left(N_{b}-1\right)\cdot PRI}\\ a_{N_{b}}\cdot e^{-j\frac{4\pi }{\lambda }\cdot \cos\theta_{2}\cos\varphi \cdot \upsilon \cdot N_{b}\cdot PRI}\\ \vdots \\ \vdots \\ \vdots \\ 0 \end{array} & \begin{array}{c} \vdots \\ \cdots \end{array} & \begin{array}{c} \vdots \\ \vdots \\ 0\\ a_{1}\cdot e^{-j\frac{4\pi }{\lambda }\cdot \cos\theta_{N}K\cos\varphi \cdot \upsilon \cdot \left(M-N_{b}-1\right)\cdot PRI}\\ \vdots \\ a_{N_{b}}\cdot e^{-j\frac{4\pi }{\lambda }\cdot \cos\theta_{N}K\cos\varphi \cdot \upsilon \cdot \left(M-1\right)\cdot PRI} \end{array} \end{array}\right)$$

The rows of $A_{M\times K}$ correspond to the different discrete sampling times. The columns of $A_{M\times K}$ express the weighted relationship of different targets and the antenna at the same time. The changes of added Doppler phase in different cross-range angles are shown in the measurement matrix. $a_{n_{b}}$ in $A_{M\times K}$ is the antenna pattern weighted coefficient, and Nb=θbθbω·PRIω·PRI is the sampling number of one beamwidth, where $\theta_{b}$ is the beamwidth and PRI is the pulse recurrence interval. For the entire imaging region, the relationship of the column and row of $A_{M\times K}$ is:

(8) $$M=K+N_{b}-1.$$

As shown by the model can be represented by Equation (7), in addition to the Doppler history, the antenna pattern is also considered in forward-looking radar imaging. Different from the SAR imaging, the antenna pattern plays the key role to reconstruct the target distribution, and the Doppler term just makes the signal model more accurate when compared with the conventional convolution model [13,14,16]. Although the resolution of the superresolution method for forward-looking imaging will be lower than the SAR technique for side-looking imaging, the superresolution method is an effective way to improve the resolution of forward-looking imaging.

## 3. Sparse Bayesian Algorithm for High Cross-Range Resolution

In this section, we employ a Bayesian estimator to reconstruct the original image domain.

### 3.1. Sparse Bayesian Superresolution Algorithm

The maximum a posteriori (MAP) estimation can be considered as a Bayesian version of maximum likelihood (ML) estimation, with prior information of the target included. The basic form of the MAP estimator is:

(9) $$\begin{array}{c} \hat{x}=\arg\;\max_{x}p\left(x|g\right)=\arg\;\max_{x}\left[p\left(g|x\right)p\left(x\right)\right] \end{array}$$

where $p\left(\cdot \right)$ represents the probability density function, $p\left(x|g\right)$ is the a posteriori probability density function, $p\left(g|x\right)$ is the likelihood probability density function (which is determined by the statistical properties of noise), and $p\left(x\right)$ is the prior information of the target scattering coefficient. The MAP estimator is equivalent to the ML estimator when there is no prior information about the target distribution.

Though many Bayesian approaches have been proposed to deal with the signal recovery problem of forward-looking superresolution imaging, most of them adopt Poisson statistics, which do not apply to the deconvolution of the motion platform. These approaches are valid for noncoherent radar, but such an imaging system has a small set of applications, and the uncorrectable assumption of the noise statistic causes the degradation of superresolution performance. In radar imaging, the statistics of noise often obey a Gaussian distribution. Meanwhile, in order to overcome the complex-valued signal recovery problem, we assume that the observation noise is statistically independent and obeys a circular complex Gaussian distribution. The likelihood probability density function (pdf) can be written as:

(10) $$p\left(x|g\right)=\frac{1}{\left(\pi \sigma^{2}\right){}^{MK}}\exp\left[-\frac{1}{\sigma^{2}}{∥g-\mathrm{Ax}∥}_{2}^{2}\right].$$

Another important task is determining how to correctly select prior information of target scatters. In [23], a sparsity promoting prior was given:

(11) $$p\left(x\right)\propto \prod_{n=1}^{NK}\exp\left[-\frac{2}{q}\left({\left|x_{n}\right|}^{q}-1\right)\right]$$

where 0<q≤1. When q=1, $p\left(x\right)\propto \exp\left(-2{∥x∥}_{1}\right)$ becomes the Laplace distribution. When q→0, the prior term becomes px∝∏n=1NK11xn2xn2. In general, the prior corresponding to a smaller one has a sharper peak at zero and provides sparser estimates in Bayesian inference. This distribution is used to express the prior information. Since the main applications of forward-looking imaging are target searching, location, and tracking, the target distribution corresponding to the whole forward-looking imaging region is independent and sparse. In addition, because noise is an important factor in the performance of deconvolution, the sparseness prior in signal processing has denoising capability [31]. The objective function can be written as:

(12) $$\begin{array}{c} p\left(g|x\right)\cdot p\left(x\right)=\frac{1}{{\left(\pi \sigma^{2}\right)}^{MK}}\exp\left[-\frac{1}{\sigma^{2}}{∥g-\mathrm{Ax}∥}_{2}^{2}\right]\prod_{n=1}^{NK}\exp\left[-\frac{2}{q}\left({\left|x_{n}\right|}^{q}-1\right)\right]. \end{array}$$

It is difficult to directly calculate the MAP solution of Equation (12). Because $\ln\left(x\right)$ is a monotonic function in terms of x, it is much easier to calculate the maximum value of $ln\left(p\left(g|x\right)p\left(x\right)\right)$ than $p\left(g|x\right)p\left(x\right)$. To this end, we use the negative logarithm operation to transform the problem as:

(13) $$\begin{array}{c} \begin{array}{c} \hat{x}=\arg\;\min_{x}\left[-\frac{1}{2}\ln\left(p\left(g|x\right)\right)-\frac{1}{2}\ln\left(p\left(x\right)\right)\right], \end{array} \end{array}$$

and the objective function is transformed into:

(14) $$\begin{array}{c} \begin{array}{c} \hat{x}=\arg\;\min_{x}\left[-\frac{1}{2\sigma^{2}}{∥g-\mathrm{Ax}∥}_{2}^{2}+\sum_{n=1}^{NK}\frac{1}{q}\left({\left|x_{n}\right|}^{q}-1\right)\right]. \end{array} \end{array}$$

In Equation (14), the noise parameter $\sigma^{2}$ is unknown and needs to be estimated. The estimation of this parameter will be discussed at the end of this section. Regardless of the unknown parameter $\sigma^{2}$, we first calculate the conjugate gradient of Equation (14) with respect to x and make it equal to zero:

(15) $$\begin{array}{c} \begin{array}{c} \nabla f\left(x\right)=\frac{1}{\sigma^{2}}A^{H}\mathrm{Ax}-\frac{1}{\sigma^{2}}A^{H}g+P^{-1}x \end{array} \end{array}$$

where $P=diag\left\{p_{1},\cdots ,p_{NK}\right\}$ and $p_{n}={\left|x_{n}\right|}^{2-q}$. Minimizing (15) by letting $\nabla f\left(x\right)=0$, the formula can be written as:

(16) $$\begin{array}{c} \left(A^{H}A+\sigma^{2}P^{-1}\right)x-A^{H}g=0. \end{array}$$

It is hard to get the optimal solution of x from Equation (16) because the matrix P is a nonlinear function of x. In order to solve this problem, we employ a heuristic approach. The coarse least-squares approximation solution $x={\left(A^{H}A\right)}^{-1}A^{H}g$ is chosen as the initial value first. Then, we construct a simple solution of x:

(17) $$\begin{array}{c} x={\left(A^{H}A+\sigma^{2}P^{-1}\right)}^{-1}A^{H}g. \end{array}$$

We then substitute the initial value of x into Equation (17) to calculate the new $x^{1}$. The initial value does not need high accuracy because the iterative strategy makes the recovery solution to the real value step by step. Next, we use the new solution $x^{1}$ to update P. The iterative expression can be given as [23]:

(18) $$\begin{array}{cc} x^{l+1} & ={\left(A^{H}A+\sigma^{2}{\left(P^{l}\right)}^{-1}\right)}^{-1}A^{H}g\\ & =P^{l}A^{H}{\left(A^{H}P^{l}A+\sigma^{2}I\right)}^{-1}g \end{array}$$

where l+1 is the iterative number, $P^{l}=diag\left\{p_{1}^{l},\cdots ,p_{NK}^{l}\right\}$, and $p_{n}^{l}={\left|x_{n}^{l}\right|}^{2-q}$. In the calculation of Equation (18), direct matrix inversion leads to high computational complexity. Therefore, we compute the iterative solution by using the conjugate gradient (CG) method. First, we denote D=P12AHσ212I and u=0σ2−12g, which makes ${\left(A^{H}\mathrm{PA}+\sigma^{2}I\right)}^{-1}g$ equivalent to ${\left(D^{H}D\right)}^{-1}D^{H}u$. Now, the problem becomes a least-squares problem, which can be solved by the CG method. Therefore, a significant speed improvement will be achieved.

### 3.2. Estimation of the Parameter $\sigma^{2}$

In practical radar signal processing, the statistical parameter $\sigma^{2}$ of the complex Gaussian noise is unknown and needs to be accurately estimated. In this paper, we propose an adaptive approach to update the estimation $\sigma^{2}$ according to the corresponding signal recovery result of Equation (18). The proposed estimation method is based on the assumption that the noise obeys a zero mean and circular complex Gaussian distribution.

The variance of the noise can be computed by the following equation because the mean of the noise is zero:

(19) $$\sigma^{2}=\frac{1}{MK}{∥g-\mathrm{Ax}∥}_{2}^{2}.$$

Since x is the target distribution that we need to estimate, we can only estimate $\sigma^{2}$ according to the coarse least-squares approximation solution, which may cause serious estimation degradation in the target distribution. Thus, we employ the iterative strategy to continue updating $\sigma^{2}$, which combines the iterative estimation results of Equation (18). After each iteration of Equation (18), we substitute the estimation results into Equation (19) to recalculate $\sigma^{2}$. The new $\sigma^{2}$ is also substituted into Equation (18) to improve the estimation accuracy. The iterative expression is then:

(20) $${\left(\sigma^{2}\right)}_{l}=\frac{1}{MK}{∥g-Ax_{l}∥}_{2}^{2}.$$

Note that this procedure assumes that the noise variance $\sigma^{2}$ used in Euqation (18) will be consistent with the estimate in Equation (20) only for the correct value. It has been shown in [35] that this is not always the case. This means that the algorithm may converge to a local minimum, giving an estimate for $\sigma^{2}$ that is not exactly correct. This should not necessarily be a problem, as long as the estimation error of $\sigma^{2}$ is not too large.

## 4. Simulations

The simulation results of point and scene targets are given to verify the performance of the proposed Bayesian deconvolution method. The superresolution performance is compared with the Poisson-based MAP algorithm because it has been shown in [19] that it provides better superresolution performance than some traditional forward-looking imaging methods.

In the simulations, the added noise obeys a zero mean and complex Gaussian distribution, and the signal-to-noise ratio (SNR) is defined as:

(21) $$\begin{array}{c} SNR=20\;\log_{10}\frac{{∥x∥}_{2}}{{∥\hat{x}-x∥}_{2}}. \end{array}$$

Note that the SNR herein is that of the cross-range echo after matched filtering in range. The forward-looking radar transmits linear frequency-modulated pulses, and can provide significant SNR gain by matched filtering. Typically, when the bandwidth of the LFM signal is 50 MHz and the pulse width is 2 μs, the match filtering can provide an SNR gain by 30 dB. When the output SNR of the radar receiver is 0 dB, the ultimate SNR of the azimuth echo can reach 30 dB. Therefore, in the following simulation, the SNR will be set to the range from 15 dB to 25 dB, which will be reasonable according to the practical case. Some main simulation parameters are given in Table 1.

### 4.1. Simulation of Point Targets

**Figure 2:**
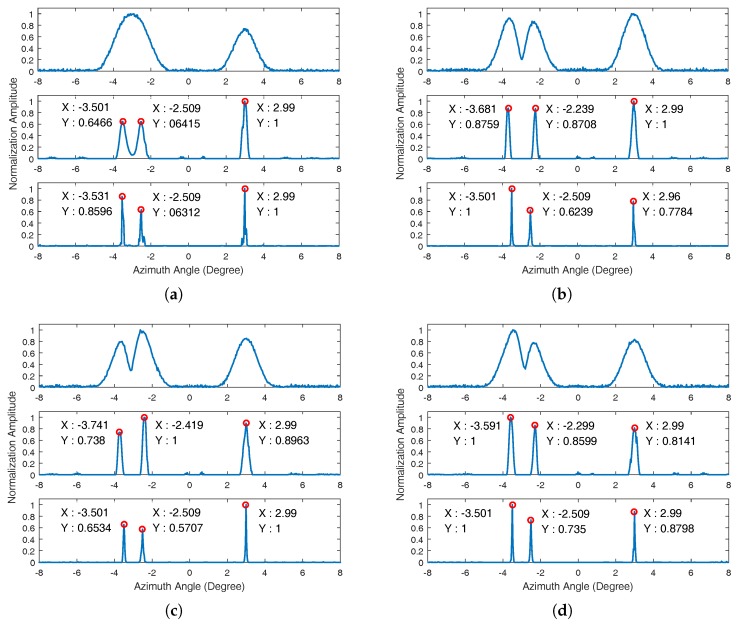
Simulation results of point target when the signal-to-noise ratio (SNR) is 25 dB. The top subfigure is the cross-range received signal after motion compensation; the middle subfigure is the superresolution result processed by the Poisson-based maximum a posteriori (MAP) algorithm; and the bottom subfigure is result processed by the proposed sparse Bayesian algorithm. (**a**) Platform velocity is 0 m/s; (**b**) platform velocity is 100 m/s; (**c**) platform velocity is 200 m/s; (**d**) platform velocity is 300 m/s.

**Figure 3:**
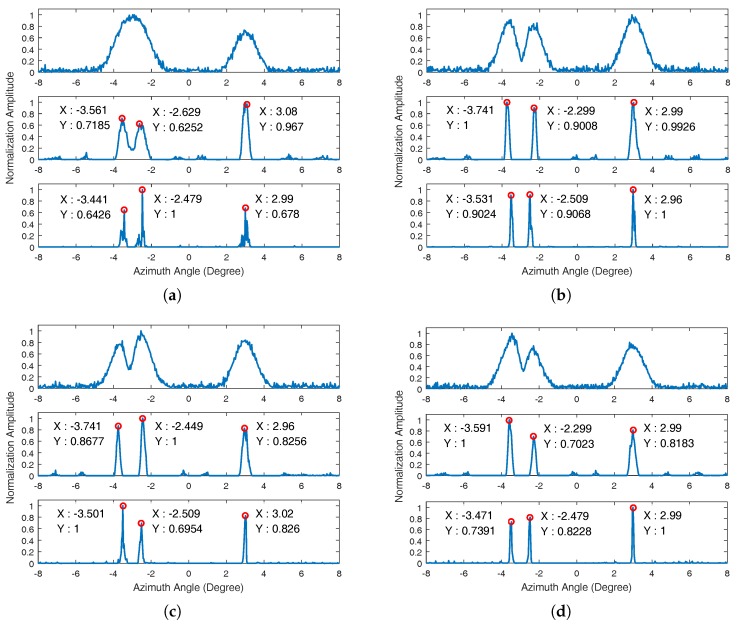
Simulation results of the point target when the SNR is 15 dB. The top subfigure is the cross-range received signal after motion compensation; the middle subfigure is the superresolution result processed by the Poisson-based MAP algorithm; and the bottom subfigure is the result processed by the proposed sparse Bayesian algorithm. (**a**) Platform velocity is 0 m/s; (**b**) platform velocity is 100 m/s; (**c**) platform velocity is 200 m/s; (**d**) platform velocity is 300 m/s.

We first consider examples of point scatterers with different platform velocities. Suppose that three targets with the same unit amplitude are located at the forward-looking region of the platform. The cross-range angles of the three targets are $-3.5^{\circ }$, ${-2.5}^{\circ }$, and $3^{\circ }$, respectively. The distance between the original position of platform and the center target is 20 km. Figure 2 and Figure 3 show the simulation results in different conditions.

Figure 2 shows the simulation results processed by the proposed sparse Bayesian algorithm and Poisson-based MAP algorithm when the SNR is 25 dB. The added noise obeys a zero mean and circular complex Gaussian distribution. We use a single cross-range signal profile to show the performance of the proposed algorithm in different platform velocities. Figure 2 includes the simulation results when the platform velocities are 0 m/s, 100 m/s, 200 m/s, and 300 m/s, respectively. The top of each subfigure shows the received cross-range signal after motion compensation. The middle and bottom of the subfigures are the simulation results processed by the Poisson-based MAP algorithm and the proposed sparse Bayesian algorithm, respectively.

Figure 2a shows that the isolated point target is sharpened and the adjacent targets are resolved by both algorithms when the platform is stationary. Although the assumption that the noise distribution obeys a Poisson distribution is different from the practical added noise, the superresolution performance of the Poisson-based MAP algorithm is still high because the SNR is high in this set of simulations. The location error of the Poisson-based MAP algorithm appears in the superresolution results with the motion platform. With the influence of the Doppler phase, the received signal of the motion platform in the cross-range dimension is not the real signal convolution. We can clearly find the shape-shifting of received signals under different motion velocities. The Poisson-based MAP algorithm is a real-valued signal deconvolution algorithm. We need to do the modulus operator before applying this method to the cross-range signal superresolution, which causes the location error. The isolated point target did not have any location error because this target did not have any overlap among other targets. However, the location error of adjacent point targets is about $0.2^{\circ }$ in motion platform simulation. For instance, the working distance is 20 km in the simulations, so the location error of the 20-km-away targets is about 70 m. This is a significant error in practical applications. This problem is overcome by the proposed sparse Bayesian algorithm. For both the stationary platform and the moving platform scenario, the proposed algorithm achieves higher target location accuracy. Besides, the proposed Bayesian superresolution algorithm has better superresolution performance than the Poisson-based MAP algorithm. It sharpened the point targets approximately four times more than the Poisson-based MAP algorithm.

Figure 3 shows the simulation results when the SNR is 15 dB. From this figure, we can find that the Poisson-based MAP algorithm has a performance degradation. First, the location error still exists, and the angle location error almost reaches $0.3^{\circ }$ when the platform velocity is 200 m/s. Besides, the phenomenon of noise amplification in the superresolution results appeared. With the decrease of SNR, the phenomenon of noise amplification will seriously influence the estimation of the target number and position. The proposed sparse Bayesian algorithm has superior superresolution performance in the low SNR condition. The noise is suppressed because the penalty term was considered in the proposed sparse Bayesian algorithm. In addition, the proposed sparse Bayesian algorithm still compressed the target about four-times more than the Poisson-based MAP algorithm in the low SNR condition. The point target simulations demonstrate the performance of the proposed sparse Bayesian algorithm.

**Figure 4:**
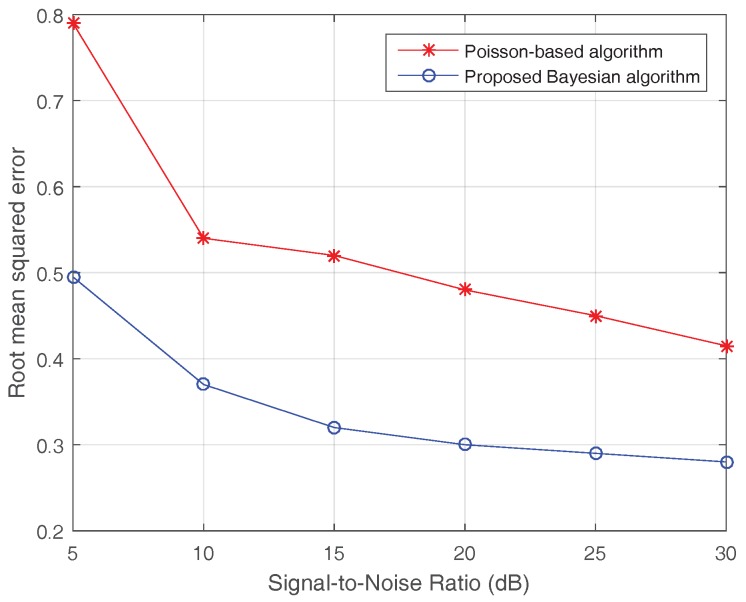
Root mean squared error (RMSE) curves of the Poisson-based MAP algorithm and the proposed sparse Bayesian algorithm.

Figure 4 shows the curves of the Poisson-based MAP algorithm and the proposed sparse Bayesian algorithm under different SNRs. Compared to the Poisson-based algorithm, the proposed algorithm obviously has a smaller RMSE and faster convergence rate. It has almost converged when the SNR is higher than 15 dB. The simulation results verify that the proposed sparse Bayesian algorithm is suitable for the practical condition of forward-looking imaging.

### 4.2. Scene Simulation

**Figure 5:**
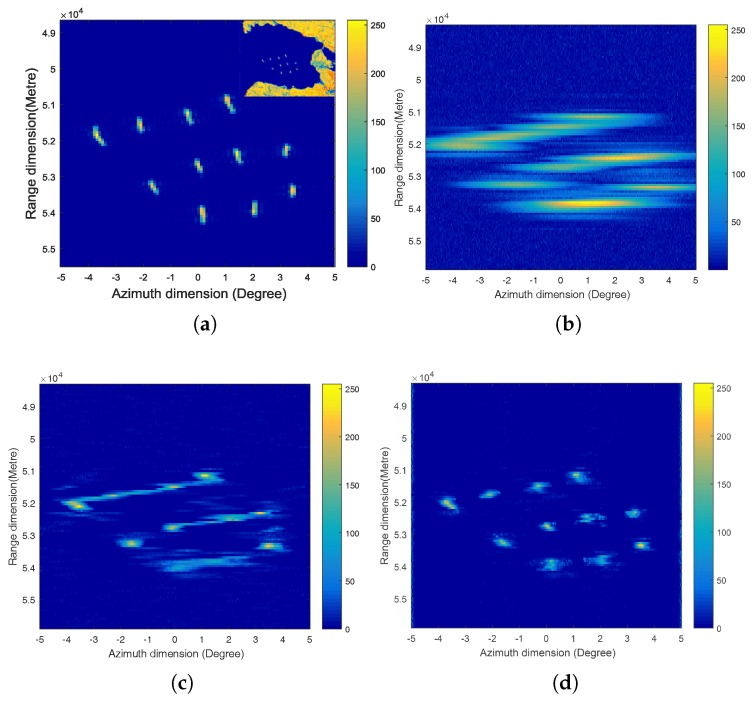
Simulation results of scene when the SNR and signal to clutter ratio (SCR) are 20 dB and 15 dB, respectively. (**a**) Original scene. (**b**) Real beam image after pulse compression and motion compensation. (**c**) Superresolution result processed by the Poisson-based MAP algorithm. (**d**) Superresolution result processed by the proposed sparse Bayesian algorithm.

This section shows the simulation results of a 2D scene containing ships. Figure 5a–d are the original scene, the real beam received signal after pulse compression and motion compensation, the superresolution results processed by the Poisson-based MAP algorithm, and the proposed sparse Bayesian algorithm, respectively. Because forward-looking imaging mainly concentrates on the applications of target searching and resolving, the original scene we consider is an area of strong scattering targets in part of an SAR image. In this simulation, the forward-looking imaging region is from $-5^{\circ }$ to $5^{\circ }$, and the working distance is 5 km. The platform velocity is 200 m/s, and the SNR is 20 dB. Because this simulation is for sea surface targets, sea clutter that obeys the *K* distribution is added in the image, and the signal-to-clutter ratio (SCR) is 15 dB. Figure 5b shows that the ships have very low cross-range resolution, and the adjacent ships cannot be distinguished in the real beam image.

In the simulation results of the two algorithms, although the performance of the proposed sparse Bayesian algorithm is influenced by the sea clutter, we can clearly find that it has better superresolution performance than the Poisson-based MAP algorithm. When some ships are in one beamwidth, the performance of the Poisson-based MAP algorithm has serious degradation. Not only are the targets hardly resolved, but severe location error exists in the processed result. The proposed algorithm avoids these phenomena, whether or not the isolated ships or the adjacent ships all have high cross-range resolution and location accuracy. We can easily obtain accurate information of the ship number and position. The scene simulation also verified that the proposed sparse Bayesian algorithm is an effective superresolution algorithm for forward-looking imaging under a cluttered background.

**Figure 6:**
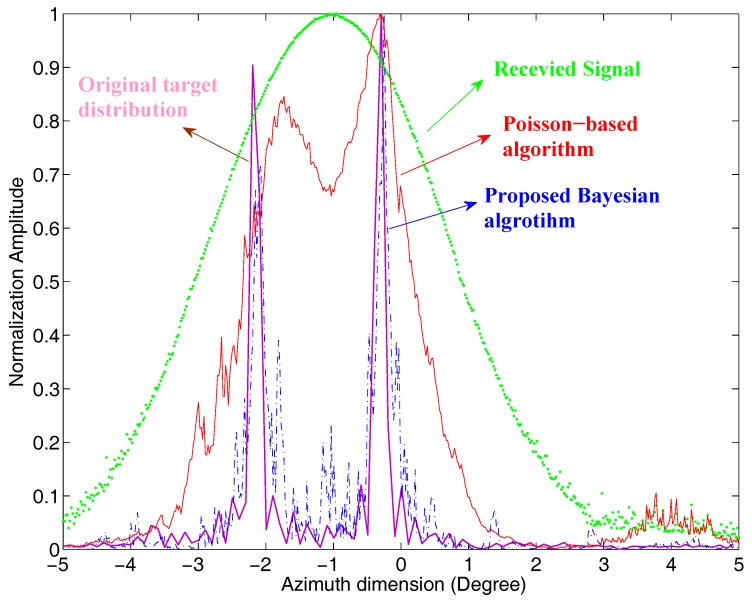
Profiles of the single range bin.

Figure 6 shows the profile of the range bin of 5.16 km of the scene simulation. The pink, green, red, and blue lines are the profiles of the original targets distribution, the real beam echo after motion compensation, the simulation results processed by the Poisson-based MAP algorithm, and the proposed sparse Bayesian algorithm, respectively. At this range, there are two targets in one beamwidth, at about $-2.2^{\circ }$ and $-0.2^{\circ }$. The proposed algorithm successfully distinguishes the two targets and causes very small location error, and the recovered cross-range width of the target almost equals the real target, which reaches a resolution improvement of 1.5-times. However, the Poisson-based algorithm has poor superresolution performance. The adjacent targets are hardly distinguished, and the target at $-2.2^{\circ }$ has very serious location error of $0.31^{\circ }$, whereas the proposed sparse Bayesian algorithm enjoys a lower location error of $0.08^{\circ }$.

## 5. Real Data Processing

**Figure 7:**
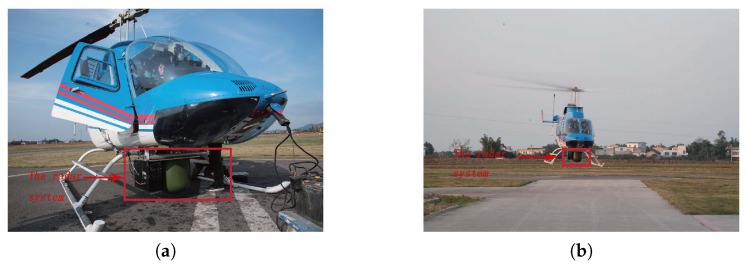
The Ka bandwidth radar system and helicopter platform. (**a**) The Ka bandwidth radar system. (**b**) The helicopter platform.

The performance of the proposed algorithm has also been proven by real measured data. Here, the measured Ka band radar data of the motion platform is used to verify the performance of the new algorithm. Figure 7 shows the installation of the Ka band radar system and a flight photo of the motion platform. From Figure 7a,b, we can find that the radar system is fixed at the bottom of the helicopter, which points to the front of the airplane heading. It irradiates the forward-looking region and records the received data.

**Figure 8:**
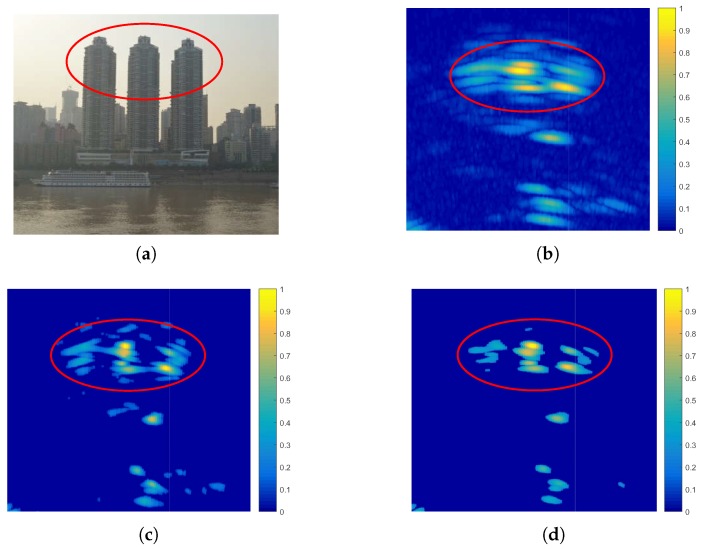
Processed results of real measured data. (**a**) The forward-looking imaging region of three buildings. (**b**) Real aperture image after pulse compression. (**c**) Superresolution result processed by the Poisson-based MAP algorithm. (**d**) Superresolution result processed by the proposed algorithm.

**Figure 9:**
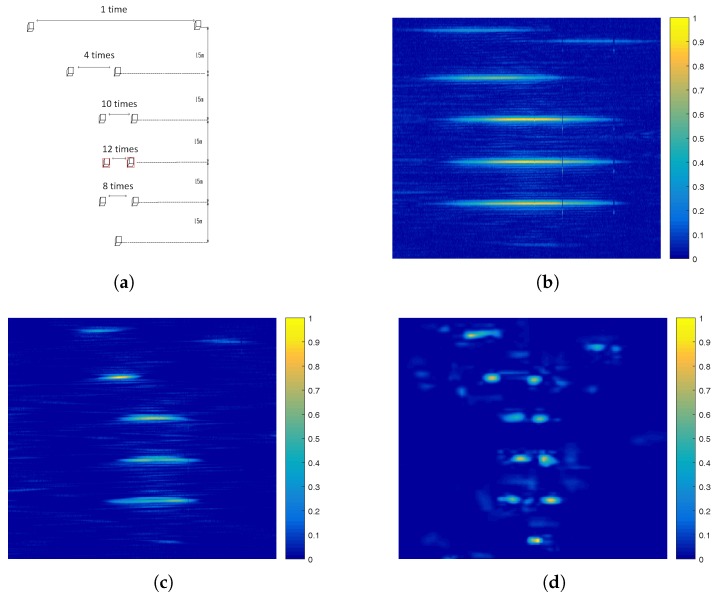
Processed results of real measured data. (**a**) The distribution of corner reflectors. (**b**) Real aperture image after pulse compression in the range dimension. (**c**) Superresolution result processed by the Poisson-based MAP algorithm. (**d**) Superresolution result processed by the proposed algorithm.

Figure 8 and Figure 9 are two real measured data, which can verify that the proposed sparse Bayesian algorithm can be used in the typical applications of the obstacle avoidance of helicopters in adverse weather and ground target location and tracing, respectively.

In the first experiment, the forward-looking imaging region of the helicopter mainly includes three buildings, which cannot be resolved in the real beam image of Figure 8b. The helicopter must spend more time passing around the buildings to avoid striking. Although the Poisson-based MAP algorithm improves the cross-range resolution, it cannot guide the pilot to across the buildings with a shortcut. The proposed sparse Bayesian algorithm significantly improves the cross-range resolution about four-times more than the Poisson-based MAP algorithm. We can clearly find the spacing among the buildings that makes us choose a better route to across the obstacles.

In the second experiment, we put some corner reflectors in specific positions to verify the ground target detection performance of the proposed algorithm. In order to determine the superresolution limitation of the proposed algorithm, the experiments employ six groups of corner reflectors which are located at different range units with different intervals, including one isolated target and five groups of adjacent targets with an interval of one-time, four-times, eight-times, 10-times, and 12-times, respectively, corresponding to the real aperture antenna beamwidth. Figure 9a shows the distribution of corner reflectors.

Figure 9 shows the processed results of real measured data. Figure 9b shows the real aperture image after pulse compression and motion compensation in the range dimension. In this experiment, the ground clutter has little affect on the imaging result because this experiment is to simulate the application of the target location of close-range and strong scattering targets. Compared with the real aperture image and superresolution results of the Poisson-based MAP approach, we can find that the proposed approach can compress the single target and resolve the adjacent targets. It improves the cross-range resolution by more than 12-times compared to the real beam echo, and the maximum location error of the corner reflectors is $0.1^{\circ }$. However, the Poisson-based MAP approach cannot resolve most of the adjacent targets. It just compress the single corner reflector about three-times as the beamwidth. In conclusion, the processed result of the proposed approach has better cross-range resolution and contour features than the real aperture and the processed result of the Poisson-based MAP approach.

## 6. Conclusions

Low cross-range resolution of the forward-looking region of the motion platform is a traditional problem of radar imaging. In order to overcome this problem, this paper proposed a signal recovery algorithm based on the Bayesian theory. Firstly, the signal model of forward-looking scanning radar in the cross-range dimension was built and rearranged as the matrix and vector expression. Then, the statistical properties of noise and target scatters were considered as the likelihood function and prior information of the Bayesian formula. The derivation process of the objective function is described in detail. Simulation results show that the proposed method significantly improves the cross-range resolution and quality of the radar image.

## Figures and Tables

**Table 1:** Simulation parameters.

Parameter	Value	Units
Carrier frequency	9.6	GHz
Band width	50	MHz
Beamwidth	3	${}^{\circ }$
Antenna scanning velocity	30	${}^{\circ }$/s
Antenna scanning area	−10 to +10	${}^{\circ }$
Pulse repetition frequency	1000	Hz
Platform height	1000	m
Incident angle	30	${}^{\circ }$
Working distance	20	km
